# Belladonna and Blethers

**Published:** 1921-12

**Authors:** 


*To the Editor of* THE HOSPITAL AND HEALTH REVIEW.

### Belladonna and Blethers.

*To the Editor of* THE HOSPITAL AND HEALTH REVIEW.

SIR,—In all the discussion which the Glasgow Corporation's case has produced, I have noticed no reference to the important point that in every public park and garden the vegetation is theoretically taboo. The smallest toddler, let alone the schoolboy, knows that by breaking that taboo he is tempting Providence and the park-keeper. It is not contended that the unlucky child who died was

**CORRESPONDENCE—(*cont.*).**

within his rights in plucking the belladonna berries which he ate. Why, then, should any public body be held liable when disaster follows the breaking of its by-laws?

If this case is to form a precedent, there is a bad time ahead for park authorities. Can anyone tell us whether an action for damages will lie in the not unlikely event of (1) the breaking of a rotten bough in a park tree causing a climbing boy to break his neck; (2) a child sitting on a wasps' nest within the park precincts? We might even go further and anticipate a day when cautious authorities will order their park-keepers to apprehend all children with cut knees and march them off for anti-tetanic treatment.—Yours faithfully,

A RATEPAYER.

